# Model for Nanopore Formation in Two-Dimensional Materials by Impact of Highly Charged Ions

**DOI:** 10.1021/acs.nanolett.2c03894

**Published:** 2022-11-18

**Authors:** Alexander Sagar Grossek, Anna Niggas, Richard A. Wilhelm, Friedrich Aumayr, Christoph Lemell

**Affiliations:** † Institute for Theoretical Physics, TU Wien, Wiedner Hauptstr. 8-10, A-1040Vienna, Austria; ‡ Institute of Applied Physics, TU Wien, Wiedner Hauptstr. 8-10, A-1040Vienna, Austria

**Keywords:** two-dimensional materials, highly charged ions, potential sputtering, nanopore formation

## Abstract

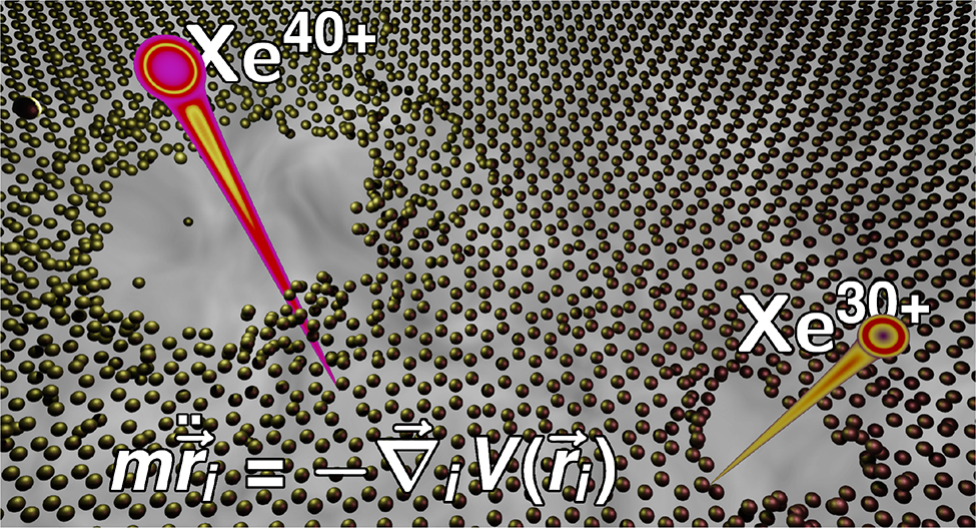

We present a first qualitative description of the atomic dynamics in two-dimensional (2D) materials induced by the impact of slow, highly charged ions. We employ a classical molecular dynamics simulation for the motion of the target atoms combined with a Monte Carlo model for the diffusive charge transport within the layer. Depending on the velocity of charge transfer (hopping time or hole mobility) and the number of extracted electrons which, in turn, depends on the charge state of the impinging ions, we find regions of stability of the 2D structure as well as parameter combinations for which nanopore formation due to Coulomb repulsion is predicted.

In order to exploit the special properties of two-dimensional materials for novel applications as nanoelectronic devices and sensors, it has become common to stack them into artificial layer systems of semimetals (e.g., graphene^1,2^), semiconductors (e.g., transition-metal dichalcogenides^3−5^), and insulators (e.g., hexagonal boron nitride^6^) as van der Waals (vdW) heterostructures.^7,8^ Tailoring the properties of heterostructures after growth requires modification techniques with single-layer precision. Highly charged ions (HCIs) are one tool to manipulate and modify 2D heterostructures with the necessary accuracy. For example, when a freestanding vdW-MoS_2_/graphene heterostructure was recently irradiated with Xe^38+^ ions, it was found that nanometer-sized pores formed only in the MoS_2_ monolayer facing the ion beam while the graphene beneath remained intact.^9^ The known high material selectivity in the deposition of potential energy of HCIs had been noticed earlier,^10,11^ but to explore its mechanisms in more detail is essential for a targeted application in the processing of vdW heterostructures on the nanoscale: e.g., for water purification^12^ or filtering CO_2_ and other gases.^13,14^ To test the response of 2D materials to the deposition of large amounts of potential (i.e., electronic) energy, experiments have been conducted in which HCIs were directed on such targets.^15,16^ A range of materials was covered from conducting single-layer graphene (SLG), for which the ejection of numerous electrons (multiple times the initial HCI charge *Q*_in_) and structural integrity was observed,^9,10,17^ to semiconducting MoS_2_, which disintegrates around the point of ion impact in combination with the emission of only a few electrons.^18^ For the latter material nanopores with a diameter of a few nanometers were observed with the mean radius depending on the initial charge state of the HCI.^11^ Recently, a more systematic study on the response of freestanding fluorinated SLG (fluorographene) was performed, confirming the strong dependence of the pore radius on the electronic properties (tunable band gap, charge mobility^19−22^) of the target.^23^

It was assumed that the effect leading to pore creation could be related to bond breaking and the electrostatic repulsion of target atoms that remain charged long enough to initiate target disintegration (potential sputtering). Without reneutralization of the impact area, the repulsive Coulomb potential would quickly exceed the binding potential of the target atoms, leading to material damage that can only be avoided if charge transport in the layer proceeds fast enough. Accordingly, the high electron mobility in SLG would enable fast charge diffusion and ensure stability of the target, while MoS_2_ disintegrates when excited by an HCI due to its electron mobility being about 3 orders of magnitude smaller.^24−27^

To investigate this hypothesis we have set up a simulation consisting of three distinct parts which are closely intertwined. (A) The motion of atoms is modeled with a MD simulation solving Newton’s equations of motion in a crystal potential and accounting for the Coulomb repulsion of positively charged ions. (B) The layer is charged due to electron transfer to the impinging projectile. (C) Positive holes left on the surface after electron transfer are distributed diffusively over the surface via stochastic hopping between neighboring lattice sites. Different charge mobilities are accounted for by the hopping time *t*_h_. While it is clear that hopping of localized charges is an inappropriate description for charge transport in conducting materials, we use the model also for SLG as a limiting case for very small hopping times: *t*_h_ → 0.

Atomic units (*e* = *m*_e_ = *ℏ* = 1 au) are used throughout the paper unless otherwise stated.

## Molecular Dynamics Simulation

**Figure 1:**
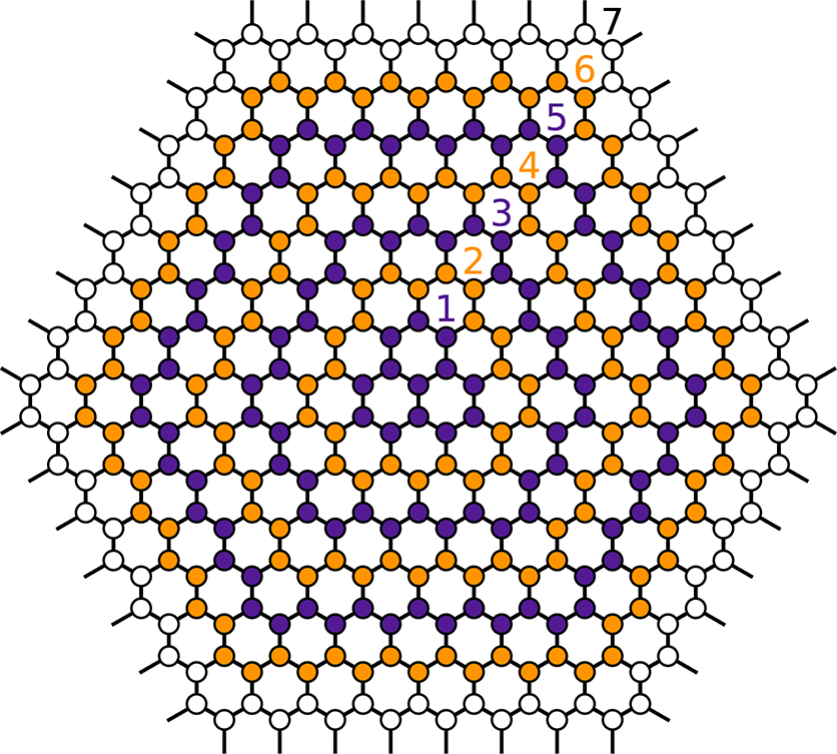
Ring structure of a simulated graphene flake. Shown here is a graphene flake with seven rings. Each colored circle represents a carbon atom. The individual rings are distinguished by their alternating colors in purple and orange. The white circles in the edge ring represent carbon atoms with positions remaining fixed throughout the simulation.

We model graphene flakes as a number of concentric rings *N*_ring_ around the impact point of the HCI on the surface (middle atom, Figure 1). The distance between neighboring carbon atoms is set to the equilibrium distance at 300 K (*d*_*CC*_ = 1.42 Å), the bond angles are 120°, i.e., a perfect honeycomb structure. Depending on the initial charge state of the projectile and the charge mobility of the target layer, the number of rings is adapted to achieve converged simulations. For example, for projectiles with a kinetic energy of 0.7 keV/amu, charge states as large as *Q*_*in*_ = 35, and a hopping time of about 5 au we find convergence of our results starting from *N*_ring_ ≥ 11 rings (∼800 atoms), which we use for most of the results presented in this letter. In our simulation atoms of the outermost ring do not move and charges hopping onto this ring are removed from the simulation and no longer contribute to Coulomb repulsion.

A variety of potentials have been used to model graphene and its thermal and mechanical properties. A popular type of potential that has been successfully used in describing 2D materials is the Stillinger–Weber (SW) potential^28^ (for other possible choices see, e.g., refs (29−31)). We use it in this simulation due to its small numerical cost while successfully reproducing the splitting of graphene grains into graphene nanoplatelets when strained.^32^

The SW potential originally developed to model silicon^28^ consists of a distance-dependent pair potential ϕ_2_ and a bond potential depending on the angle between any three atoms ϕ_3_:
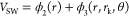
1with
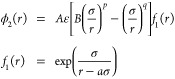
and
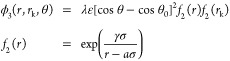


The parameters for the potential used in this work (Table 1) are optimized to reproduce the mechanical properties of graphene.^32^

**Table 1:** Parameters for the Stillinger–Weber Potential (eq 1) for Graphene Platelets^a^

*A*	5.8341
*B*	0.6022
*p*, *q*	4, 0
γ, *a*	1.2, 1.8
θ_0_	120°
σ	1.28 Å (2.419 au)
ε	5.44 eV (0.2 au)
λ	40

a From ref (32).

**Figure 2:**
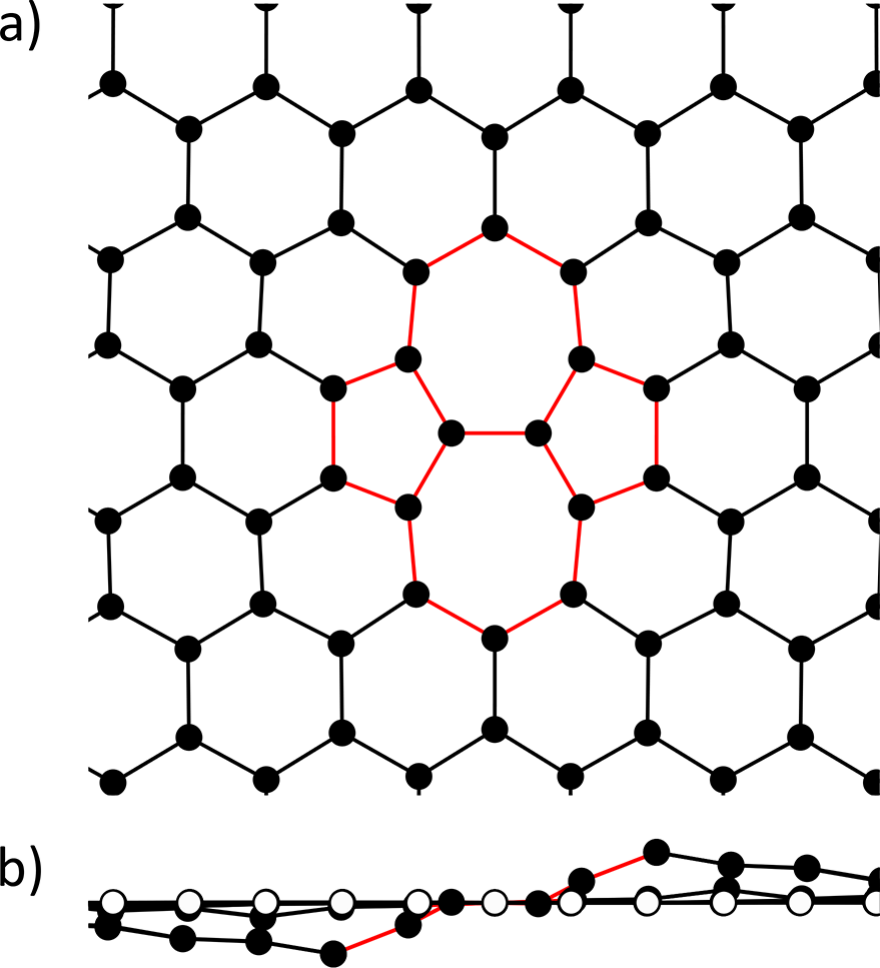
Simulated Stone–Wales defect in graphene flake:^33^ (a) lattice irregularity; (b) buckling in plane.

Using these parameters, not only the ground-state lattice can be stabilized but also typical lattice defects such as the Stone–Wales defect (combination of rings of seven and five atoms;^33^Figure 2).

From the curvature of the potential at its minimum we calculate the oscillation frequencies in-plane and out-of-plane to determine the randomized starting positions and velocities for our MD simulations for all carbon atoms in the graphene sheet at 300 K from a microcanonical ensemble.

We solve Newton’s equations of motion using a fourth-order variable time step Runge–Kutta integrator to model the motion of carbon atoms in the potential landscape defined by eq 1 and by additional unshielded positive hole charges localized on the target atoms after electron transfer from the impact area to the impinging projectile. Note that the potential (eq 1) is not altered in our simulation due to charge depletion. Furthermore, kinetic momentum transfer from the projectile onto lattice atoms is currently still neglected, as it induces mainly localized defects, e.g., a Stone–Wales defect (Figure 2) or single vacancies, too small to be detected in typical experimental setups.^11,34^ We follow the motion of the atoms until the lattice is free of charges, i.e., charges have migrated to the “bulk” outside the outermost ring. After conclusion of the simulation, we compare the individual binding potential of each atom with its kinetic energy. Even if only a single particle’s kinetic energy is larger than its binding potential, the target is counted as restructured. Again, such small changes in the lattice structure (single vacancies) are hard to detect in experiments. Application of this criterion will therefore result in an estimate of the lower bound of the stability of SLG.

## Electron Transfer to Projectile

Various studies have been devoted to the simulation of electron transfer to slow HCIs in front of solids and 2D layers (e.g., refs (10 and 35−37)), establishing the extremely short time scale on which the neutralization of the projectiles happens. As the positively charged ion approaches the surface, it will extract electrons from the target to excited projectile states that will eventually cascade down to less excited states in Auger or radiative decay processes. For the initial projectile charge states considered here (*Q*_in_ = 30–40) a total of 90–150 electrons are estimated to be extracted from the target (sum of electrons emitted to vacuum and stabilized on the projectile^18^).

We simulate electron transfer based on a simplified version of the classical-over-the-barrier (COB) model.^38^ Accordingly, electron transfer starts at a critical distance given by
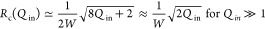
2with *W* being the work function of the material. In the current simulation we are not interested in details of the neutralization process of the HCI or the electron-emission statistics, only in the total number of electrons extracted from the impact area by the HCI. Therefore, we simplify the COB model and determine only the average distance between subsequent electron transfers Δ*r* from the change of *R*_c_ as a function of the charge state
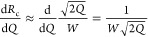
3
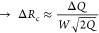
4for Δ*Q*/*Q* ≪ 1. Starting with an electron transfer at *R*_c_(*Q*_in_), we stochastically determine the distance to the next transfer as  with  being a uniformly distributed random number .

As the electron is captured into a highly excited state of the HCI, its screening of the nucleus is incomplete. Additionally, processes such as Auger deexcitation lead to the emission of electrons intermittently increasing the charge state of the HCI. Therefore, one transferred electron will on average lead to a reduction of the charge state Δ*Q* < 1. We use Δ*Q* as a free parameter to achieve an overall electron extraction along the trajectory of the ion as measured in experiments.^18^ E.g., for Xe ions with a kinetic energy of 0.7 keV/amu and *Q*_in_ = 35, an average amount of 120 electrons is extracted.^18^ For this exemplary case we find the best agreement for Δ*Q* ≈ 0.29. This is in reasonable agreement with previous full simulations of the COB model for HCI approaching metal surfaces.^39^

In our model, electrons can be transferred only from the central atom and atoms of ring 1 due to the strong distance dependence of the transfer rates^40^ and each atom can supply a maximum of two electrons. Once an atom has lost both electrons, no further electron transfer to the HCI is possible unless the positive hole charges move along the layer by diffusive transport, thus reneutralizing the vicinity of the impact point and allowing for further electron transfer. With this limit to the central atoms of the flake, we are able to reproduce the small amount of extracted electrons for 2D layers with small charge mobility (MoS_2_^18^) without changing the parameter Δ*Q*, lending credence to the overall layout of the electron-transfer simulation.

## Hopping Conduction

Transport of (localized positive) charges on the surface is accounted for by a simulation of diffusive transport by stochastic hopping of charges to neighboring lattice sites. Migration of a charge is simulated in two steps. First, a random number determines if a transfer happened during a time step Δ*t* depending on the hopping time (average time between subsequent transfers) *t*_*h*_, , and then the new position of the charge is chosen randomly from any of the three neighboring lattice sites. If the selected site is already doubly charged, the transfer is suppressed. This part of the simulation has a single free parameter, the hopping time *t*_h_ determining the diffusion constant  of the unbiased hopping process which, in turn, is directly proportional to the charge carrier mobility μ via the Einstein relation, *eD* = μ*k*_B_*T*, with the electron (hole) charge *e*, the Boltzmann constant *k*_B_, and the absolute temperature *T*. Electron mobilities in 2D materials range from up to 200000 cm^2^/(V s)^41^ for annealed and suspended single-layer graphene (typical values are 1 order of magnitude smaller) down to the mobility of monolayer MoS_2_ with less than 80 cm^2^/(V s).^42^ Actual velocities used in this simulation have been deduced from the band structure of the materials, accounting for the effective masses of the holes. Charge hopping continues for target atoms set in motion but still close to other target atoms, thus leading to a large fraction of neutral sputtered atoms. Currently, interactions of the hole charges with the HCI are still neglected, underestimating the diffusion speed. Furthermore, the resulting conductivity has been shown to depend not only on the charge density but also on the distribution of charges on the surface.^43^

In summary, during a time step of duration Δ*t* various processes may happen. (A) Atoms in the lattice are accelerated due to the crystal and Coulomb potentials (B) Electron transfer from the target to the HCI occurs if at least one of the atoms in the impact region (i.e., within the first carbon ring) is less than doubly charged. (C) Localized charges on the surface migrate between atoms due to hopping conduction, affecting, in turn, A and B.

We have performed a large set of calculations varying the parameters *Q*_in_ and *t*_h_ (hopping time or, equivalently, charge mobility of the target). For each combination of *Q*_in_ and *t*_h_ close to 1500 impacts were simulated.

**Figure 3:**
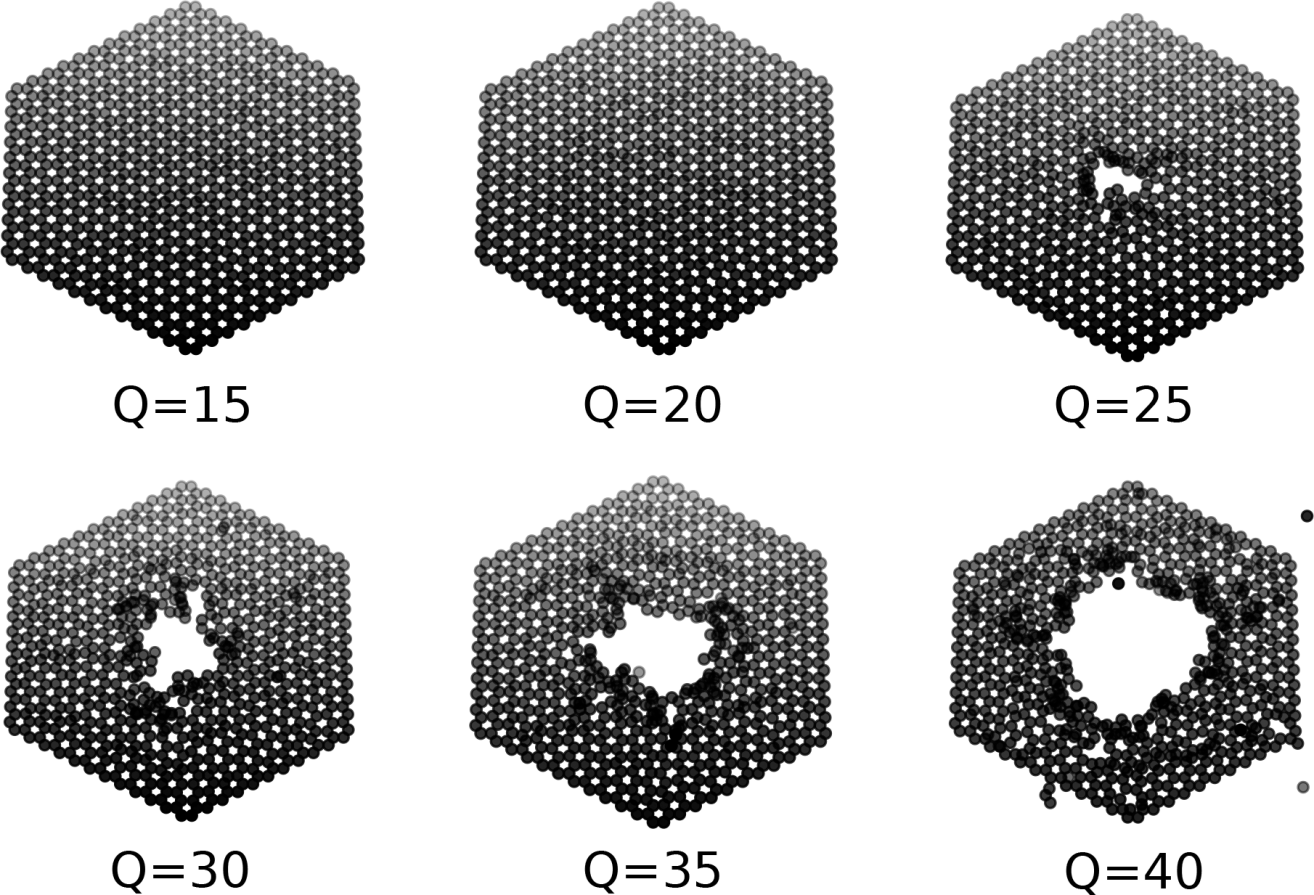
Restructuring of carbon layers due to impact of a highly charged ion with initial charges ranging from *Q*_*in*_ = 15–40 for a fixed hopping time of *t*_*h*_ = 7 au (∼0.17 fs). The threshold for pore formation at this charge mobility is around *Q*_in_ = 25.

For a fixed hopping time of *t*_h_ = 7 au Figure 3 shows the final positions of carbon atoms after conclusion of the simulations for different values of *Q*_in_.

As observed in experiments, the size of the nanopores increases as a function of *Q*_in_. For this particular *t*_h_, pore formation sets in at *Q*_in_ ≈ 25 where we observe restructuring of the target only in a fraction of our simulations (∼25%). For higher charge states (e.g., *Q*_in_ = 40) the efficiency for pore formation approaches 100%.

**Figure 4:**
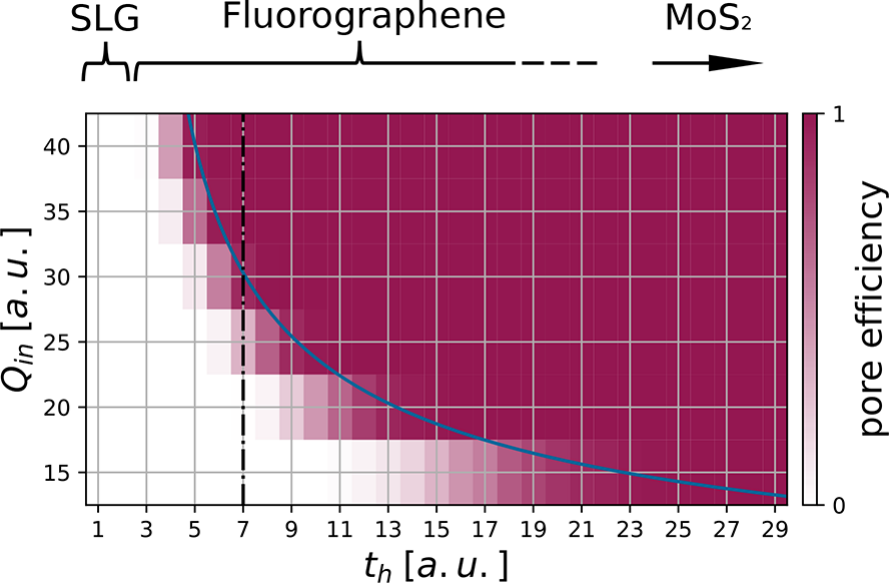
Phase diagram of the simulated interactions of HCIs with different initial charge *Q*_in_ with a graphene flake of 11 rings with varying charge mobilities. Initial charges *Q*_in_ were simulated in steps of 5 elementary charges and hopping times *t*_h_ in steps of 1 atomic unit. Combinations of *Q*_in_ and *t*_h_ that leave the layer unaltered are shown in white, combinations with a 100% probability for restructuring in purple. Color shadings indicate parameter combinations for which both intact and restructured layers have been found at the conclusion of the simulation. Results shown in Figure 3 are located along the vertical dot-dashed black line at *t*_h_ = 7. The blue line indicates the border at which average pore sizes reach a diameter of 1 nm.

The results of our simulations are summarized in a phase diagram (Figure 4) with a relatively small transition area separating regions of stability (white area with small *Q*_in_ and *t*_h_) and pore formation (purple area with large *Q*_in_ and *t*_h_). Shaded regions indicate that both intact and altered surfaces have been found at the conclusion of the simulation. It has been shown that fluorographene becomes increasingly susceptible to pore formation with increasing fluorination.^23^ The fluorination alters the band structure of SLG, introducing a band gap and reducing the charge mobility.^19−22^ Increasing levels of fluorination reduce the charge mobility in fluorographene, likely aiding its susceptibilty to pore formation. At the top of Figure 4 we relate increasing hopping times to increasing degrees of fluorination (reduced mobility). While HCI-induced pores were never seen on SLG^10,17^ (semimetal with very small *t*_h_), increasing fluorination (increasing *t*_h_) leads to pore formation with increasing probability. Irradiation of MoS_2_ with its very small charge mobility (outside the range of *t*_h_ covered in this work) always results in pore formation even for moderate initial charge states of the HCI.^9,11^ Below a critical hopping time *t*_c_ ≈ 2.5 au, surface restructuring was never observed in our simulation irrespective of the initial charge of the HCI. The transition region between stable and unstable regions loosely follows a  dependence. Note that the threshold found in this work provides a lower limit for the pore formation in 2D materials due to the charge-interaction effects neglected in our (unbiased) hopping simulation.

**Figure 5:**
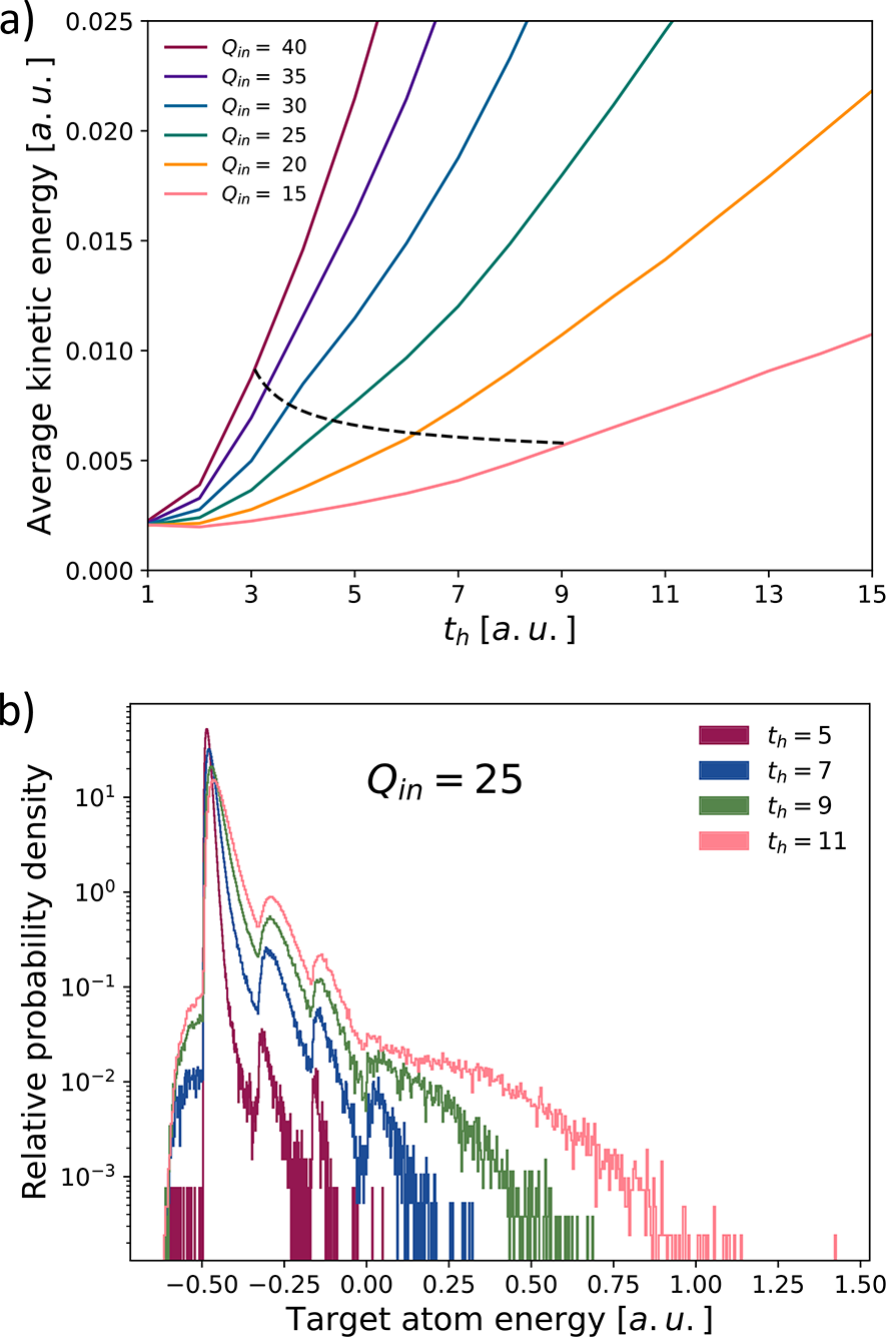
Energy distribution of target atoms at the conclusion of the simulations. (a) Average energy per particle. The approximate kinetic energy threshold for pore formation for different incident charge states *Q*_in_ as a function of the hopping time is indicated by the dashed line. Note that in the threshold region not every impact of an HCI leads to target restructuring. (b) Energy distribution of individual target atoms for *Q*_in_ = 25 and hopping times in the threshold region. Atoms bound in the crystal lattice have negative energies. The three distinct peaks in the distribution indicate the relative probability densities of the number of atoms that still have three, two, and one lattice neighbors. One au in time corresponds to 24.2 attoseconds, one au in energy to 27.2 eV.

Finally, we have also analyzed the energy distribution of sputtered particles at the end of our simulation. For this analysis we concentrated on the region of the phase diagram (Figure 4) in or close to the transition region where we expect the least influence of size effects (choice of *N*_ring_), the charge-transport model, or the crystal potential on the final energy distribution of the particles. As can be expected due to the wide discrepancy of atom and hole velocities, most of the released carbon atoms have small kinetic energies and do not carry any charges. Once an atom is charged and starts to move due to Coulomb repulsion, its charge may still for a long time (on the time scale of the hole) be transferred to a nearby atom, leaving the starting atom without further acceleration. Furthermore, atoms are accelerated predominantly in the plane of the layer which transfers a considerable fraction of their kinetic energy on nearby atoms, thus “heating” the lattice (Figure 5a). A different heating mechanism (strong electron–phonon coupling not present in our model) has been proposed recently to explain the velocity distribution of Mo atoms released from MoS_2_ supported on Au and SiO_2_.^44^ In the threshold region in our study (shaded area in Figure 4) only a few atoms are sputtered (positive energies in Figure 5b) with small kinetic energies leaving behind atoms still bound in the crystal lattice (at negative energies) but with one or two neighboring atoms missing with binding energies of around −0.3 and −0.15 au, respectively. Due to the high stability of the SLG lattice, the target can be heated up locally to rather high temperatures before releasing individual atoms. Of course, this strong binding can be overcome by Coulomb forces if the hole charges are not diffusing away from the impact area quickly enough. The largest part of the energy, however, is still absorbed by the lattice and not by single sputtered particles.

To summarize, we have shown that the pore formation in clean and fluorinated graphene layers due to the impact of a highly charged ion can be qualitatively modeled with a molecular dynamics simulation for the motion of atoms in combination with Monte Carlo charge hopping for the charge conduction within the target layer. Based on the charge mobility of 2D materials, this model is able to reproduce the dependence of the pore diameter on the initial charge state of the impinging projectile or the reduction of the number of electrons extracted from materials with small charge mobility. Only for single-layer graphene, a material with high mobility, pore formation was not observed irrespective of *Q*_in_. For smaller charge mobilities disintegration of the layer (pore formation) due to repulsive forces can be expected above a threshold charge state . We find an approximate dependence on the square root of the charge mobility, . This result is stable with respect to changes in the depth of the crystal potential or the parameter governing the neutralization sequence, Δ*Q* (not shown).

This gives confidence that our phase diagram properly captures the qualitative dependence of pore formation in HCI–2D layer interactions on the initial charge state of the projectile and the relevant material parameter of the 2D structure, its charge mobility. Our results can therefore serve as guidelines when looking for 2D materials that are susceptible to nanopore formation by slow, highly charged ion impact.
